# Antiviral activity of marine sulfated glycans against pathogenic human coronaviruses

**DOI:** 10.1038/s41598-023-31722-5

**Published:** 2023-03-23

**Authors:** Mary Zoepfl, Rohini Dwivedi, Seon Beom Kim, Michael A. McVoy, Vitor H. Pomin

**Affiliations:** 1 Department of Chemistry, Virginia Commonwealth University, Richmond, VA 23284 USA; 2 Department of BioMolecular Sciences, University of Mississippi, University, MS 38677 USA; 3 Department of Food Science and Technology, College of Natural Resources and Life Science, Pusan National University, Miryang, 50463 Republic of Korea; 4 Department of Pediatrics, Virginia Commonwealth University, Richmond, VA 23298 USA

**Keywords:** Drug discovery, Microbiology, Diseases

## Abstract

Great interest exists towards the discovery and development of broad-spectrum antivirals. This occurs due to the frequent emergence of new viruses which can also eventually lead to pandemics. A reasonable and efficient strategy to develop new broad-spectrum antivirals relies on targeting a common molecular player of various viruses. Heparan sulfate is a sulfated glycosaminoglycan present on the surface of cells which plays a key role as co-receptor in many virus infections. In previous work, marine sulfated glycans (MSGs) were identified as having antiviral activities. Their mechanism of action relies primarily on competitive inhibition of virion binding to heparan sulfate, preventing virus attachment to the cell surface prior to entry. In the current work we used pseudotyped lentivirus particles to investigate in a comparative fashion the inhibitory properties of five structurally defined MSGs against SARS-CoV-1, SARS-CoV-2, MERS-CoV, and influenza A virus (IAV). MSGs include the disaccharide-repeating sulfated galactan from the red alga *Botryocladia occidentalis*, the tetrasaccharide-repeating sulfated fucans from the sea urchin *Lytechinus variegatus* and from the sea cucumber *Isostichopus badionotus*, and the two marine fucosylated chondroitin sulfates from the sea cucumbers *I. badionotus* and *Pentacta pygmaea*. Results indicate specificity of action against SARS-CoV-1 and SARS-CoV-2. Curiously, the MSGs showed decreased inhibitory potencies against MERS-CoV and negligible action against IAV. Among the five MSGs, the two sulfated fucans here studied deserve further attention since they have the lowest anticoagulant effects but still present potent and selective antiviral properties.

## Introduction

Emerging viral infections that give rise to impactful pandemics are historic threats to humankind. The past two decades, for instance, have seen the emergence of three coronaviruses, severe acute respiratory syndrome coronavirus 1 (SARS-CoV-1) in 2003^1,2^, Middle East respiratory syndrome coronavirus (MERS-CoV) in 2013^3^, and severe acute respiratory syndrome coronavirus 2 (SARS-CoV-2) in 2019^4^, besides two IAVs, H1N1 in 2009 and H7N9 in 2013^5^. SARS-CoV-2 led to the devastating COVID-19 pandemic that was not only impactful on global health but also disrupted economies and social behaviors^6–8^. Numerous other viruses, such as HIV, have also emerged in the twentieth century^9^. As each new virus appears, challenges of developing new vaccines and therapeutics also arise. In this regard, the discovery or development of broad-spectrum antivirals have the potential to fight several problems at the same time.

**Figure 1:**
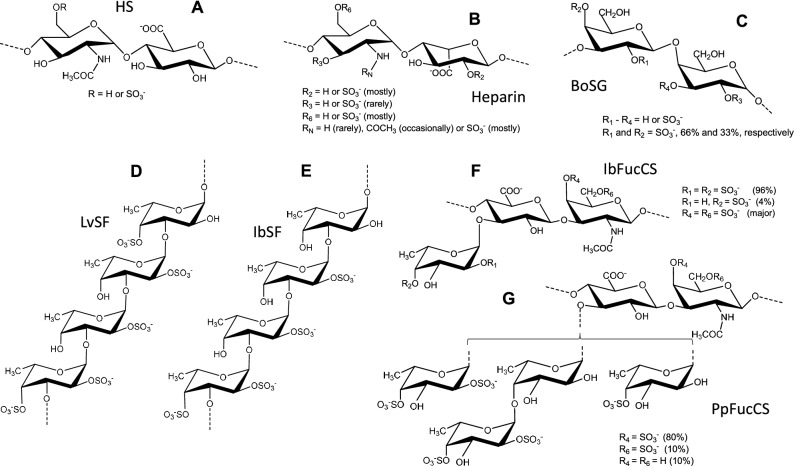
Structural representations of HS, heparin and the five MSGs studied. (**A**) HS is composed of [-4)-N-acetylglucosamine-(α1-4)-glucuronic acid-(α1-]. (**B**) Heparin is composed of [-4)-N,6-disulfated-glucosamine-(α1-4)-2-sulfated-iduronic acid-(α1-] with additional modifications as indicated in the panel. (**C**) BoSG is composed of [-3)-galactose-(β1-4)-galactose-(α1-]_n_ with variable sulfation patterns as indicated in the panel. (**D**) LvSF is composed of [-3)-2,4-disulfated-fucose-(α1-3)-2-sulfated-fucose-(α1-3)-2-sulfated-fucose-(α1-3)-4-sulfated-fucose-(α1-]_n_. (**E**) IbSF is composed of [-3)-2,4-disulfated-fucose-(α1-3)-2-disulfated-fucose-(α1-3)-2-sulfated-fucose-(α1-3)- fucose-(α1-]_n_. (**F**) IbFucCS is composed of {-4)-[ fucose-(α1-3)]-glucuronic acid-(β1-3)-N-acetylgalactosamine-(β1-} with variable sulfation patterns as indicated. (**G**) PpFucCS is composed of {-β3)-N-acetylgalactosamine-X-(β1-4)-glucuronic acid-[(3–1)-Y]-(1-}, where X = 4S (80%), 6S (10%) or non-sulfated (10%), Y = α-Fuc2,4S (40%), Fuc2,4S-(α-1–4)-α-Fuc (30%), or α-Fuc4S (30%), and S = SO_3_^−^, as indicated in the panel.

A common strategy to develop a broad-spectrum antiviral is to target a shared molecular player or mechanism commonly used by multiple viruses. Following this rationale, molecular interactions of viral proteins with surface glycans of host cells is a common event seen in infections of many viruses, including SARS-CoV-1^10^, SARS-CoV-2^11^, MERS-CoV^12^, and influenza viruses^13^. Anionic glycans such as heparan sulfate (HS) (Fig. 1A), a sulfated glycosaminoglycan (GAG), and sialic acids are common sugars that often serve as co-receptors that mediate attachment of virus particles to host cells prior to entry^14^.

Polycationic peptides, small molecules, metal coordination complexes, and glycan mimetics have been used to inhibit viral attachment^15–20^. Small peptide fragments of viral entry proteins can bind to HS in place of the virion, thereby inhibiting viral infection^16,17^. However, these peptides often only inhibit the virus from which they are derived. Alternatively, glycan mimetics bind to the virion in place of HS, allowing broad activity against viruses that utilize HS for attachment^21^. Unfortunately, GAG mimetics with high and variable molecular weights (MWs) and complex structural heterogeneity are often poor candidates for therapeutic applications. In contrast, marine sulfated glycans (MSGs) are endowed with more regular structures than mammalian GAGs^22–25^ and therefore offer better prospects as antivirals. Additionally, marine polysaccharides are readily available in nature, nontoxic, inexpensive, and biocompatible, making them attractive natural products for antiviral development^26^. However, sulfated glycans, regardless of their classes as GAG analogs or MSGs, often have undesirable anticoagulant properties^23,27–30^ and this poses a challenge to rationalize the clinical applications of these sugars as potential antivirals. Two strategies exist to overcome this issue. One relies on the occasional identification of naturally occurring sulfated glycans with low or negligible anticoagulant activities but significant antiviral activities^22^. The other relies on chemical modifications that selectively reduce the anticoagulant activity of the sulfated glycans while maintaining their antiviral properties^31^.

In this work, we used pseudotyped lentivirus-based virus-like particles (VLPs) to assess the antiviral activities of five MSGs against SARS-CoV-1, SARS-CoV-2, MERS-CoV, and IAV. Comparative analyses were performed among the five different MSGs and heparin, an HS mimetic widely exploited in research^32^ whose structure is primarily composed of the disaccharide repeating units of [-4)-N,6-disulfated-glucosamine-(α1-4)-2-sulfated-iduronic acid-(α1-] (Fig. 1B). The five MSGs were: a sulfated galactan isolated from the red alga *Botryocladia occidentalis* (BoSG)^33^, two sulfated fucans (SFs) isolated from the sea urchin *Lytechinus variegatus* (LvSF)^34^ and the sea cucumber *Isostichopus badionotus* (IbSF)^35^, and two fucosylated chondroitin sulfates (FucCSs) isolated from the sea cucumbers *I. badionotus* (IbFucCS)^36^ and *Pentacta pygmaea* (PpFucCS)^22^.

BoSG is composed of the disaccharide repeating unit [-3)-2,4-disulfated-galactose-(β1-4)-2,3-disulfated-galactose-(α1-]_n_ in which sulfation patterns may vary in percentage but never in position (MW > 100 kDa) (Fig. 1C)^29^. LvSF is composed of the tetrasaccharide-repeating unit of [-3)-2,4-disulfated-fucose-(α1-3)-2-sulfated-fucose-(α1-3)-2-sulfated-fucose-(α1-3)-4-sulfated-fucose-(α1-]_n_ (MW ∼90 kDa) (Fig. 1D)^29^. IbSF is composed of the tetrasaccharide-repeating unit of [-3)-2,4-disulfated-fucose-(α1-3)-2-disulfated-fucose-(α1-3)-2-sulfated-fucose-(α1-3)- fucose-(α1-] (MW ∼100 kDa) (Fig. 1E)^35^. IbFucCS is composed of the trisaccharide-repeating unit {-4)-[ fucose-(α1-3)]-glucuronic acid-(β1-3)-N-acetylgalactosamine-(β1-} in which sulfation patterns may vary in percentage but never in position (MW ∼75 kDa) (Fig. 1F)^36^. PpFucCS is composed of {→ 3)-β-N-acetylgalactosamine-X-(1 → 4)-β-glucuronic acid-[(3 → 1)-Y]-(1 →}, where X = 4S (80%), 6S (10%) or non-sulfated (10%), Y = α-Fuc2,4S (40%), α-Fuc2,4S-(1 → 4)-α-Fuc (30%), or α-Fuc4S (30%), and S = SO_3_^−^ (MW ∼10–60 kDa) (Fig. 1G)^22^. Overall BoSG, PpFucCS, and IbFucCS are more heterogenous in sulfation pattern than LvSF and IbSF, which are chemically defined due to their regular composition of repetitive oligosaccharide building blocks^22–25^. Given the comparable structural variation of these MSGs, such as monosaccharide and backbone composition (galactose-based in BoSG, fucose-based in LvSF and IbSF, and heterepolysaccharides within disaccharide repeating backbone units in heparin, IbFucCS, and pFucCS), sulfation patterns (at N2, O2, O3, and O4 positions), variable composing oligosaccharide lengths (disaccharide units in heparin and BoSG, trisaccharide units in IbFucCS and PpFucCS, and tetrasaccharide units in LvSF, IbSF, and PpFucCS) and different MW distributions (ranging from ~ 15 to > 100 kDa), relationships between structure and antiviral activity can be raised.

## Materials and methods

### Cell culture

HEK-293T human embryonic kidney cells (ATTC CRL-3216) and HeLa human adenocarcinoma epithelial cells (ATTC CCL-2) were purchased from American Type Culture Collection. HEK-293T-hACE2 (HEK-293T cells expressing human angiotensin-converting enzyme 2) were purchased from BEI Resources (BEI NR-52511). HeLa-DPP4 (HeLa cells expressing dipeptidyl peptidase 4) were a gift from Linghang Peng and David Namazee^37^. Cells were cultured at 37 °C in a 5% CO_2_ atmosphere using Dulbecco’s Modified Eagle Medium supplemented with 10% fetal bovine serum, 50 U/mL penicillin, 50 mg/mL streptomycin, and 29.2 mg/mL L-glutamine (DMEM, all from Life Technologies).

### Compound isolation and purification

BoSG, LvSF, and PpFucCS were purified as previously described^22,33,34^. Sea cucumber *I. badionotus* was obtained from Gulf Specimen Lab (Gulf of Mexico, Florida Keys). IbSF and IbFucCS were isolated from *I. badionotus* following a slightly modified protocol reported earlier^35,36^. BoSG, LvSF, IbSF, PpFucCS, and IbFucCS were dissolved in water at a stock concentration of 3 mg/mL. Heparin sodium was purchased from Acros Organics, USA (Lot #B0146868; 150 IU/mg) and dissolved in water at a stock concentration of 1 mg/mL.

### Production of pseudotyped VLPs

VLPs were produced using the system developed by Crawford et al*.*^38^ with minor modifications as described previously^31^. Briefly, HEK-293T cells were cotransfected with cocktails that each contained four plasmids required for lentiviral particle production (pHAGE-CMV-Luc2-IRES-ZsGreen-W (BEI cat.# NR-52516), HDM-Hgpm2 (BEI cat.# NR-52517), HDM-tat1b (cat.# NR-52518), and pRC-CMV-Rev1b (BEI cat.# NR-52519) and a fifth plasmid encoding entry/fusion mediating glycoproteins from different viruses. Plasmid pNAHA (Addgene #44169) was used to produce VLPs pseudotyped with hemagglutinin (HA) and neuraminidase from avian IAV H7N1^39^, while plasmids pcDNA3.3_CoV1_D28 (Addgene #170447), pGBW-m4137383 (Addgene_#149541), or pCDNA3.3_MERS_D12 (Addgene #170448), were used to produce VLPs pseudotyped with spike proteins from SARS-CoV-1, SARS-CoV-2 (Wuhan strain), or MERS-CoV, respectively. VLPs in the transfected cell culture supernatants were filtered with 0.45 µm filters and stored at − 80 °C. Stocks were tittered by applying serial dilutions to appropriate target cells (HeLa-DPP4 cells for MERS VLPs, HEK-293T-hACE2 cells for SARS-CoV-1 and SARS-CoV-2 VLPs, and HEK-293T cells for IAV VLPs) in 96-well plates and after 48 h visually counting the frequency of transduced cells expressing green fluorescent protein (GFP) using a Nikon Eclipse TS100 inverted UV microscope and measuring total GFP signal in each well using a BioTek Synergy HT Multi-Mode plate reader.

### Inhibition of VLP transduction

Appropriate target cells (HEK-293T-hACE2 for VLPs pseudotyped with SARS-CoV-1 or SARS-CoV-2 spike, HeLa-DPP4 for VLPs pseudotyped with MERS-CoV spike, or HEK-293T for VLPs pseudotyped with HA) were cultured in black-wall/clear-bottom 384-well plates until confluent. Triplicate wells were then treated for one h with three-fold serial dilutions of MSGs or heparin in DMEM ranging from 400 µg/mL to 6.7 ng/mL before addition of 100 transducing units/well of VLPs. After incubation for 48 h relative fluorescence units (RFU) of GFP fluorescence in each well were quantified using a BioTek Synergy HT Multi-Mode plate reader. Prism 5 software (Graphpad) was used to determine 50% effective concentration (EC_50_) values as the inflection points of best-fit four-parameter curves for RFU (means of triplicate data) versus log glycan concentration. Graphical representations were normalized to % maximum RFU.

### Cytotoxicity

Replicate HEK-293T-hACE2, HeLa-DPP4, or HEK-293T cell cultures were prepared simultaneously with those used to determine inhibition of VLP transduction and were treated with the same glycan concentrations but were not exposed to VLPs. After 48 h cell viability was determined by removing 30 µL of culture media from each well, adding 30 µL of CellTiter-Glo® reagent (Promega), incubation for ten min. at room temperature, and measuring relative light units (RLU) using a BioTek Synergy HT Multi-Mode Microplate reader.

### Time of addition and treatment/removal studies

Confluent monolayers of appropriate target cells (described above) in black-wall/clear-bottom 384-well plates were treated with ~ 100 transducing units/well VLPs. MSGs or heparin were added to a final concentration of 150 µg/mL to triplicate wells one h before, at the time of, and 3, 6, 12, 24, or 48 h post infection. Untreated wells served as controls. GFP fluorescence was quantified 48 h post-transduction as above and percent maximum RFUs were plotted versus time of glycan addition relative to VLP addition using Prism 5 software. For treatment/removal studies HEK-293T-hACE2 monolayers in 96-well plates were treated with 150 µg/mL heparin or MSGs for 1 h, washed three times with media, then exposed to VLPs (~ 200 transducing units/well). After 48 h representative micrographs were taken with a Nikon Eclipse TS100 Inverted UV microscope.

### Coagulation assays

Although the coagulation assays were generally performed following the protocols described in^28^, more specific details of the sulfated glycans used in this work can be found in previous publications of our group^22,31^. More specifically, the aPTT was performed by incubating 90 μL of plasma with 10 μL of varying concentrations of polysaccharides at 37 °C for 3 min. 100 μL of aPTT reagent was then added to the above mixture and incubated for 5 min at 37 °C. Clotting time was measured immediately following the addition of 100 μL 0.025 M CaCl_2_. The aPTT readout was measured in seconds. Unfractionated heparin (180 IU/mg) was used as a positive control. The measurements were performed on an Amelung Coagulometer KC4A. Sulfated glycans were assayed for their serpin-mediated inhibitory activity (AT and HCII) against IIa and Xa using effective concentrations of 10 nM of AT or HCII, 2 nM of IIa or factor Xa, and 0–100 μg/mL of sulfated glycans in 100 μL of TS/PEG buffer (0.02 M Tris/HCl, 0.15 M NaCl, and 1.0 mg/mL polyethylene glycol 8000, pH 7.4) as reported earlier^40^. Sulfated glycans (10 μL) at varying concentrations were dispensed into 96-well microtiter plates followed by the addition of 40 μL AT (25 nM) or HCII (25 nM). Fifty μL of IIa (4 nM) or Xa (4 nM) was added last to initiate the reaction. The plate was then immediately incubated at 37 °C for 1 min, followed by addition of 25 μL of chromogenic substrate S-2238 for IIa or CS–11(32) for factor Xa. Absorbance was measured at 405 nm for 300 s at 15 s intervals. Wells without sulfated glycans served as controls and the means of IIa/Xa activities in the controls were considered as 100%. The residual activity in treated wells was calculated relative to that observed in the case of control wells. Heparin (180 IU/mg) was used in all the assays as a positive control while DS (CS-B) was also used as a positive control only in the HCII/IIa system.

## Results

### MSGs inhibit spike-mediated entry of SARS-CoV-1 and SARS-CoV-2 pseudotyped VLPs

**Figure 2:**
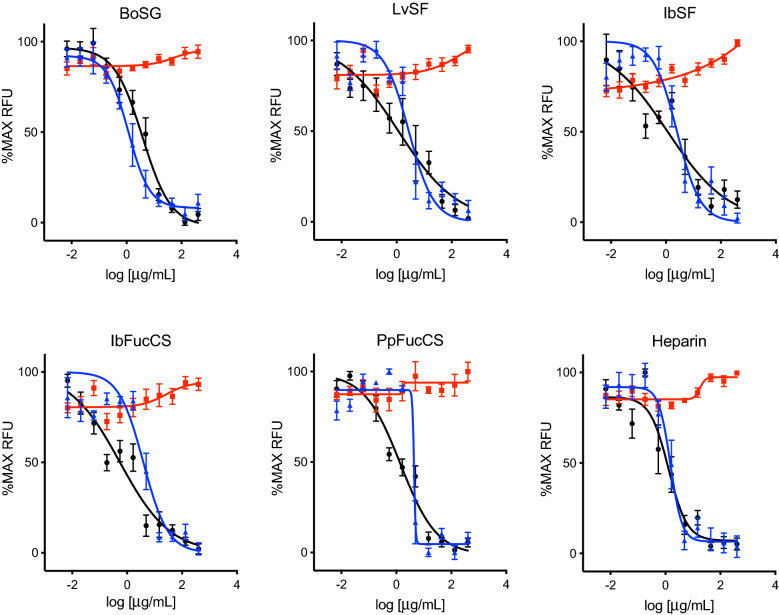
Antiviral activity of MSGs against SARS-CoV-1 and SARS-CoV-2. Anti-SARS-CoV-1 (blue) or anti-SARS-CoV-2 (black) activities were measured by incubating HEK-293T-hACE2 cell monolayers in 384-well plates with MSGs for one h, adding VLPs pseudotyped with spike from SARS-CoV-1 or SARS-CoV-2, and measuring GFP 2 days after infection. Cytotoxicity (red) was measured in replicate uninfected cultures treated for 2 days using the CellTiter-Glo® assay. Data were normalized to % maximum RFU and represent means of three independent experiments ± standard deviations.

Entry of both SARS-CoV-1 and SARS-CoV-2 is dependent on interactions between their respective spike glycoproteins with both GAGs and angiotensin converting enzyme 2 (ACE2) present on the target cell surface. Heparin is known to inhibit entry of both SARS-CoV-1 and SARS-CoV-2 by binding to spike and competitively inhibiting spike-GAG interactions^22,31,41,42^. To determine if MSGs have similar anti-SARS-CoV-1/SARS-CoV-2 activities, spike-mediated entry was modeled using lentiviral VLPs encoding a GFP expression cassette and pseudotyped with spike proteins from each virus. VLP entry was quantitated by measuring GFP fluorescence levels resulting from transduction of ACE2-expressing HEK-293T-hACE2 cells. Antiviral activities of MSGs were then assessed as the ability to inhibit GFP expression by blocking VLP entry and subsequent transduction. As shown in Fig. 2, all five MSGs inhibited GFP expression following transduction by SARS-CoV-1 or SARS-CoV-2 pseudotyped VLPs. Inhibitory potencies, determined as EC_50_ values calculated from the GFP levels, demonstrated that all five MSGs have activities against both viruses in the low µg/mL range, like heparin (Table 1). All five MSGs as well as heparin exhibited no significant cytotoxicity for HEK-293T-hACE2 cells even at the highest concentration tested of 400 µg/mL (Fig. 2), precluding accurate determination of their cytotoxic TC_50_ activities. Consequently, for each glycan the selectivity index (SI), or ratio of cytotoxicity to inhibitory potency (TC_50_/EC_50_), was estimated by assigning TC_50_ = 400 µg/mL and reporting SIs as greater than the resulting TC_50_/EC_50_ ratios (Table 1).

**Table 1:** EC_50_ activities^a^ and SIs^b^ for glycan inhibition of spike-mediated VLP entry.

*Glycans*	*SARS-CoV-1*		*SARS-CoV-2*		*MERS-CoV*	
	EC_50_	SI	EC_50_	SI	EC_50_	SI
Heparin	1.67 ± 0.21	> 239.3	2.11 ± 1.44	> 189.6	~ 400	> 1
BoSG	2.50 ± 0.39	> 159.7	2.00 ± 0.79	> 200	83.9 ± 14.1	> 4.7
LvSF	3.13 ± 0.81	> 127.8	3.99 ± 0.54	> 100.3	35.4 ± 9.4	> 11.2
IbSF	3.35 ± 0.94	> 119.1	3.75 ± 0.87	> 106.7	44.6 ± 9.1	> 8.9
IbFucCS	3.37 ± 0.99	> 118.6	1.07 ± 0.85	> 373.8	86.7 ± 11.2	> 4.6
PpFucCS	3.50 ± 0.77	> 114.3	1.64 ± 0.78	> 243.9	123.0 ± 19.7	> 3.3

^a^Glycan concentration (µg/mL) that reduces GFP signal by 50% following transduction of HEK-293T-hACE2 cells (SARS-COV-1 and SARS-CoV-2) or HeLa-DPP4 cells (MERS-CoV) with VLPs pseudotyped with spike proteins from the indicated viruses; data are means of three independent experiments ± standard deviations.

^b^TC_50_/EC_50_, assigning TC_50_ = 400 µg/mL for all glycans.

### MSGs weakly inhibit entry of VLPs pseudotyped with MERS-CoV spike and lack activity against VLPs pseudotyped with IAV HA

**Figure 3:**
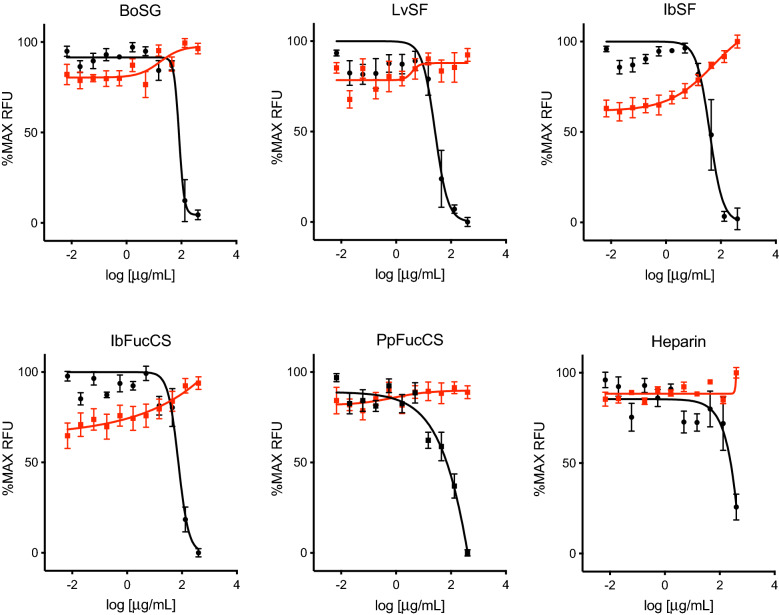
Antiviral activity of MSGs against MERS-CoV. Anti-MERS-CoV activity (black) and cytotoxicity (red) were measured as described in Fig. 2, except using MERS-CoV spike-pseudotyped VLPs and HeLa-DPP4 cells.

In contrast to SARS-CoV-1 and SARS-CoV-2, MERS-CoV does not use ACE2, but instead relies on spike interactions with dipeptidyl peptidase 4 (DPP4) for entry^43^. To determine if MSGs have anti-MERS-CoV activities, VLPs pseudotyped with MERS-CoV spike were generated and VLP entry was quantitated as GFP fluorescence following transduction of DPP4-expressing HeLa-DPP4 cells. Compared to SARS-CoV-1 and SARS-CoV-2, MSGs and heparin were considerably less active against MERS-CoV spike-mediated entry, with reductions in GFP only evident at concentrations of 100 µg/mL or above (Fig. 3 and Table 1).

**Figure 4:**
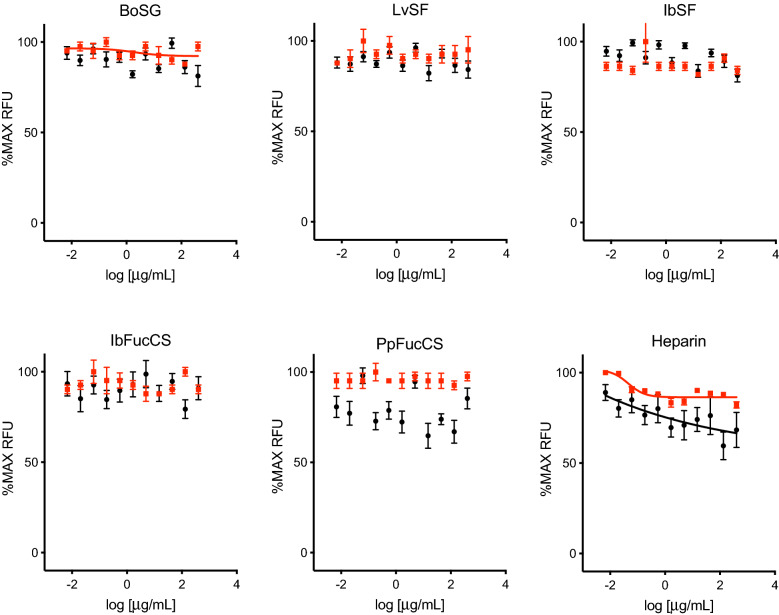
Antiviral activity of MSGs against IAV. Anti-IAV activity (black) and cytotoxicity (red) were measured as described in Fig. 2, except using IAV HA-pseudotyped VLPs and HEK-293T cells.

The above results suggested that MERS-CoV entry is only partially dependent on interactions between MERS-CoV spike and cell surface HS-containing proteoglycans. Indeed, previous reports have suggested that, like IAV, MERS-CoV entry involves spike binding to sialic acids on cell surface glycoconjugates^12^. To confirm that MSGs do not interfere with sialic acid-dependent viral entry, VLPs pseudotyped with HA from IAV were generated and entry was quantitated as GFP fluorescence following transduction of HEK-293T cells. All five MSGs as well as heparin exhibited no inhibition of GFP expression following IAV VLP transduction even at 400 µg/mL, the highest concentration tested (Fig. 4). As with HEK-293T-hACE2 cells, all five MSGs as well as heparin were nontoxic on HeLa-DPP4 and HEK-293T cells at the highest concentration tested of 400 µg/mL (Figs. 3, 4).

### Time of addition studies are consistent with MSGs blocking VLP entry

**Figure 5:**
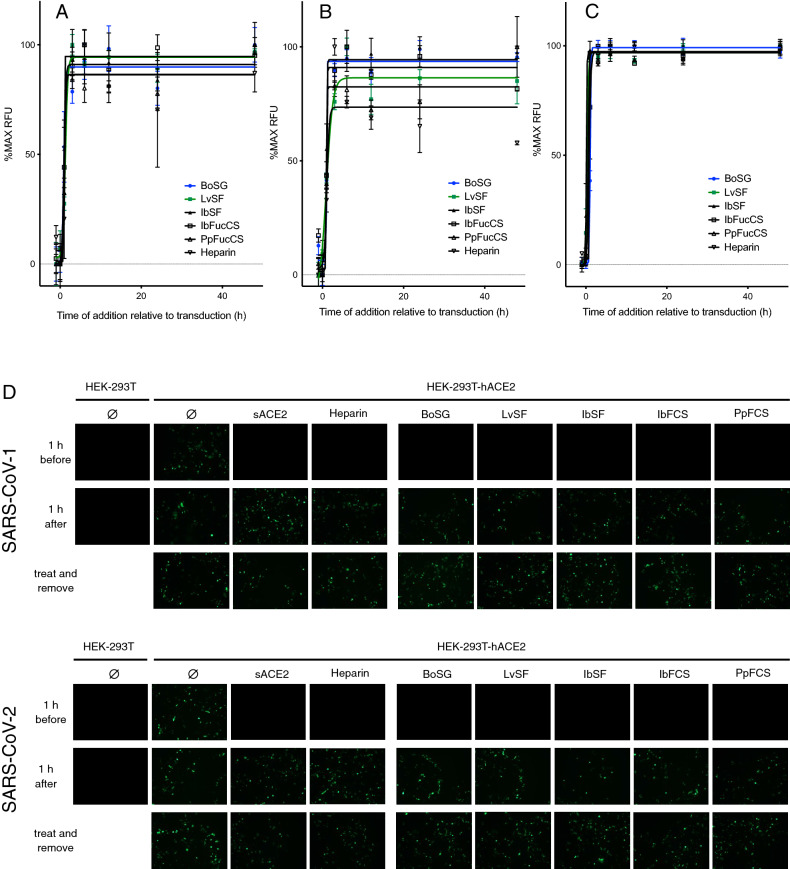
Time of addition studies. (**A**–**C**) Confluent HEK-293T-hACE2 monolayers were treated with 150 µg/mL heparin or MSGs 1 h before, concurrent with, or at various times after addition of VLPs pseudotyped with spike proteins from SARS-CoV-1 (A), SARS-CoV-2 (B), or MERS-CoV (C). GFP expression was quantified 2 days after VLPs were added. Data are means of triplicate wells ± standard deviations. (**D**) Confluent HEK-293T or HEK-293T-hACE2 monolayers were treated with medium (Ø), 300 µg/mL soluble ACE2 (sACE2), or 150 µg/mL heparin or MSGs either 1 h before or 1 h after addition of VLPs pseudotyped with spike proteins from SARS-CoV-1 or SARS-CoV-2. Representative fluorescent images were captured after incubation for 48 h. *Treat and remove* indicates replicate cultures that were treated as above for one h and then washed three times with medium prior to addition of VLPs.

Like heparin, the proposed mechanism of MSG antiviral activity is through competitive inhibition of virion spike interactions with cell surface GAGs, thus preventing virion attachment and entry. To further support this mechanism of action, time of addition studies were conducted by adding heparin or MSGs at various time points before or after addition of SARS-CoV-1 or SARS-CoV-2 VLPs to HEK-293T-hACE2 cells or addition of MERS-CoV VLPs to HeLa-DPP4 cells. Like heparin, all five MSGs only inhibited GFP expression when present prior to or very shortly after VLP addition to target cell cultures (Fig. 5A–C). Moreover, while transduction was completely blocked when SARS-CoV-1 or SARS-CoV-2 VLPs were added to cultures already containing 150 µg/mL heparin or MSGs, inhibition was lost when treated cultures were washed to remove heparin or MSGs prior to adding the VLPs, or when VLPs were added to cells one h prior to heparin or MSGs (Fig. 5D). These results are consistent with MSGs binding to VLPs rather than to cells to preclude VLP/cell attachment. Lastly, the ability of soluble ACE2 to inhibit SARS-CoV-1 and SARS-CoV-2 VLP transduction of HEK-293T-hACE2 cells but not MERS-CoV VLP transduction of HeLa-DPP4, the failure of SARS-CoV-1 and SARS-CoV-2 VLPs to transduce HEK-293T cells which do not express ACE2, and the failure of MERS-CoV VLPs to transduce HeLa cells which do not express DPP4, confirmed that entry of SARS-CoV-1 and SARS-CoV-2 VLPs is ACE2-dependent while entry of MERS-CoV VLPs is DPP4-dependent (Fig. 5D and data not shown).

### Compared to heparin, MSGs have reduced anticoagulant properties

Heparin and MSGs were assayed in two classes of anticoagulant experiments: (i) aPTT assays in which the overall impact of the sulfated glycan is measured, and (ii) serpin-dependent assays (AT-mediated inhibition of factors Xa and IIa, and HCII-mediated inhibition of factor IIa) using purified co-factors in which specific mechanisms of action were measured. Based on the overall activities of the tested compounds shown in Table 2, three major classes of polysaccharides were noted: (i) heparin, the positive control having potent effects; (ii) BoSG, IbFucCS, and PpFucCS having moderate effects; and (iii) SFs LvSF and IbSF having negligible effects. For instance, heparin shows an overall anticoagulant effect of 180 IU/mg as seen in the aPTT assay, while the BoSG/FucCSs and SFs were in the ranges of 30–50 and 3–10 IU/mg, respectively. The serpin-mediated anticoagulant actions of all sulfated glycans were directly proportional to IU/mg values observed in the aPTT assays. The preferential activities of MSGs towards the HCII-IIa system has been noted and explained previously^27,30^.

**Table 2:** Anticoagulant properties of heparin and MSGs.

*Glycans*	*aPTT* (IU/mg)^a^	*AT/IIa*	*HCII/IIa*	*AT/Xa*	References
		IC_50_ (μg/mL)			
Heparin	180.0	0.08	0.07	0.005	^31^
BoSG	21.1	0.89	0.55	0.23	^31^
LvSF	3.0	> 100	> 100	> 100	^27^
IbSF	10.0	16.1	10.6	> 100	^22^
IbFucCS	50.0	3.44	0.48	11.4	^22^
PpFucCS	29.0	4.71	0.50	11.2	^22^

^a^Values calculated using a parallel of unfractionated heparin with activity of 180 IU/mg as standard curve.

## Discussion

The anti-SARS-CoV-2 activity of BoSG, LvSF, IbSF, IbFucCS, and PpFucCS have been previously described using a pseudotyped baculoviral system^22,31,44^. The results demonstrate that these two FucCS are equally the most active of the MSGs, followed by BoSG, heparin, and IbSF, which were comparable to LvSF, the least active compounds (Table 1). This echoes what was previously reported, albeit with different EC_50_s, which is reasonable considering the two different pseudotyping systems.

Time of addition and treatment/removal studies (Fig. 5) are consistent with a mechanism in which MSGs inhibit virion attachment by binding to spike and thereby competitively inhibiting spike from interacting with cell-surface HS. This mechanism is further supported by previously published studies that used surface plasmon resonance spectroscopy to demonstrate that MSGs can bind with high affinity to various coronaviral spike protein variants and competitively inhibit heparin binding^22,31,44^. While we have not directly examined MSGs for potential virucidal effects, virucidal effects of MSG are rare in general^45^ and the mechanism by which heparin and other sulfonated compounds inhibit SARS-CoV-2 specifically has been reported to be non-virucidal^46^. Moreover, the fact that transduction by IAV VLPs is not sensitive to inhibition by heparin or MSGs demonstrates that inhibition is dependent on the specific viral entry glycoprotein used to pseudotype the VLPs and is not a consequence of non-specific virucidal effects (*e.g.*, structural disruption of the virion) or post-entry effects on reverse transcription, integration, or GFP expression.

The antiviral activities of the five MSGs were very similar against SARS-CoV-1 and very close to the same inhibitory range (1–4 μg/mL range) against SARS-CoV-2 (Table 1). Considering the similarities between spike proteins of SARS-CoV-1 and SARS-CoV-2, this result is also reasonable^11,47^. PpFucCS was the least active while BoSG was the most active against SARS-CoV-1. All MSGs were slightly less active than heparin against SARS-CoV-1 (Table 1).

However, the entire series of MSGs and heparin were much less effective against MERS-CoV, although MSGs were significantly more active than heparin (Table 1). This may be consistent with MERS-CoV spike’s affinity for HS, which is reduced compared to that of SARS-CoV-1 and SARS-CoV-2^47^. This is also in line with the differing glycan-dependence between the three coronaviruses and that polyanionic glycans are not favorable for anti-MERS-CoV activity since none of the MSGs and heparin were highly active against this virus. Table 1 shows EC_50_s in the range of tens to even hundreds of µg/mL.

It is interesting to observe that, although not as highly active as against SARS-CoV-1 and SARS-CoV-2, the MSGs showed a broad range of EC_50_ values (from 35.4 to 123.0 µg/mL) in MERS inhibition while heparin was the least active compound (Table 1). Curiously, the two low-anticoagulant SFs LvSF and IbSF were the most active among all five MSGs (Table 1). This observation illustrates structural specificity for the anti-MERS activity of MSGs and points out that the two SFs, LvSF and IbSF, with homogeneous backbones, regular sulfation patterns, and linear chains may be bearing a more active structural motif within their structures, as opposed to the other MSGs (FucCSs and sulfated galactan) with lower anti-MERS activity (Table 1).

Interestingly, the MSGs as well as heparin showed negligible anti-IAV activity (Fig. 4). This was the only non-coronavirus enveloped virus tested in this work. It is possible to hypothesize that the structural requirement for the glycans on the host cells are different among the coronavirus spike proteins and IAV HA^13^, in which sialic acid, as opposed to the role of HS in coronaviral infectivity^11^, plays a key role in IAV infectivity^14^. This lack of activity against IAV may also be due to an incomplete broad-spectrum antiviral activity as seen previously. For instance, a highly sulfated exopolysaccharide, p-KG03, from *Gyrodinium impudicum* strain KG03 (highly sulfated homopolysaccharide of galactose conjugated with uronic acid and sulfate groups) has incomplete broad spectrum antiviral activity against encephalomyocarditis virus and IAV but not influenza B virus^48,49^. This was also seen with sulfated polysaccharides studied by Baba et al*.* which were active against herpes simplex virus, human cytomegalovirus, vesicular stomatitis virus, Sindbis virus, and human immunodeficiency virus, but not against adenovirus, coxsackievirus, poliovirus, or reovirus^50^. Nonetheless, it is curious to see that some marine sulfated polymers such as spirulan^51^, alginate^52^, and fucoidan^53^ can exhibit anti-IAV activity. One possible reason for inconsistencies in anti-IAV activities is that HAs from different IAV subtypes vary significantly at the amino acid level. For example, Skidmore et al*.* found that heparin potently inhibits VLPs pseudotyped with HA subtype 5^54^, which shares only 43% amino acid sequence identity with the HA subtype 7 used in our studies. These findings, together with the lack of activity of the sulfated glycans currently studied here, clearly indicate structural specificity of sulfated glycans in IAV inhibition.

Across the three coronaviruses, MSGs maintained their mechanism of action as described previously for human cytomegalovirus and adenovirus^55^. MSGs inhibited GFP expression resulting from transduction by VLPs pseudotyped with spike proteins from SARS-CoV-1, SARS-CoV-2, and MERS-CoV only if cells were treated prior to VLP addition (Fig. 5). Washing experiments suggested that MSGs bind to virion components to inhibit attachment. MSGs are likely binding coronavirus spike protein, as supported by published results using surface plasmon resonance and molecular modeling^22,31^.

In terms of structure–activity relationships, it is generally thought that the biological activities of MSGs (*e.g.*, anticoagulation) are directly related to their MWs^30,56,57^. This did not hold true, however, as PpFucCS had the best anti-SARS-CoV-2 activity and the lowest MW. This was also observed by Dwivedi et al., who postulated that the anti-SARS-CoV-2 activity of PpFucCS may rely on special structural features, such as the branching α-Fuc-2,4S units (Fig. 1F), that compensate for its shorter backbone^22^. The sulfation levels, patterns, and monosaccharide types have also been suggested to influence viral-glycan interactions^58^. Interestingly, comparable antiviral activities of the two holothurian FucCS, PpFucCS and IbFucCS, may relate to structural similarities as both are mainly composed of branching α-Fuc-2,4S units in their structures (Fig. 1G,F). Of note, BoSG was slightly less active than the FucCSs despite its larger sulfation content and different monosaccharide type (Fig. 1C). Both sulfated fucans (IbSF and LvSF) were less active than the other MSGs but had similar antiviral activities. Although both are homopolymers of α-fucose units, they have slightly different sulfation patterns in their repeating tetrasaccharide: LvSF has one 4-sulfated (Fig. 1D) while this same unit in IbSF is not sulfated (Fig. 1E), giving LvSF a slightly higher negative charge density^35,59^. In all we can conclude that no single specific feature of the MSGs underlies their antiviral properties, but in fact, this depends ultimately on a combination of various structural features.

While heparin and its derivatives have excellent antiviral activity against several viruses, heparin is a potent anticoagulant activity (Table 2) and is associated with hemorrhage and heparin-induced thrombocytopenia^58,60^. MSGs have variable anticoagulant activity in comparison (Table 2). FucCSs are well known anticoagulants, targeting primarily the serpin heparin cofactor II, but also the tenase complex^22,61,62^. PpFucCS has moderate anticoagulant activity, lower than those of both heparin and IbFucCS^22^. BoSG has moderate anticoagulant activities (Table 2), and moderate anti-SARS-CoV-2 activity, but its anti-SARS-CoV-2 activity can be selectively separated from the anticoagulant property through size fractionation^31^. Both SFs have naturally negligible anticoagulant activities^27,35^. Although these two SFs are not the most active MSGs against coronaviruses, their low anticoagulant properties make these MSGs special candidates in future investigations of potential anti-coronaviral agents.

## Conclusions

In this study we demonstrated the antiviral nature of five MSGs having defined chemical features against pathogenic coronaviruses. While this series of MSGs have similar antiviral activities against both SARS-CoV-1 and SARS-CoV-2, they had significantly decreased activity against MERS-CoV. This may indicate the drastic difference between the spike proteins of these coronaviruses and merits further study. None of the MSGs were active against IAV, demonstrating selectivity and corroborating the sialic acid-dependence of IAV entry. The main downside of clinically developing the MSGs as novel antiviral candidates is the residual and common anticoagulant properties of this class of molecules. In this regard, the marine SFs LvSF and IbSF have the weakest anticoagulant properties yet retain potent antiviral effects. This factor makes the marine SFs LvSF and IbSF potential candidates for further clinical investigations of new broad-spectrum antiviral agents.

## Data Availability

The datasets generated and/or analyzed during the development of this current study can be shared from the corresponding authors upon reasonable request.
